# Ethanol Extract of Pomegranate (*Punica granatum*) Peel in Increasing the Expression of Caspase-3 in DSS-Induced Mice

**DOI:** 10.1155/2021/4919410

**Published:** 2021-12-02

**Authors:** Kusmardi Kusmardi, Lyanna Azzahra Baihaqi, Ari Estuningtyas, Nurhuda Sahar, Hadi Sunaryo, Aryo Tedjo

**Affiliations:** ^1^Department of Anatomic Pathology, Faculty of Medicine, Universitas Indonesia, Jl. Salemba Raya 6, Jakarta, Indonesia; ^2^Drug Development Research Center (DDRC Cluster, IMERI, Faculty of Medicine), Jakarta, Indonesia; ^3^Human Cancer Research Center (HCRC Cluster, IMERI, Faculty of Medicine) Universitas Indonesia, Jl. Salemba Raya 6, Jakarta, Indonesia; ^4^Faculty of Medicine, Universitas Indonesia, Jl. Salemba Raya 6, Jakarta, Indonesia; ^5^Department of Pharmacology and Therapeutic, Faculty of Medicine, Universitas Indonesia, Jl. Salemba Raya 6, Jakarta, Indonesia; ^6^Department of Biology, Faculty of Medicine, Universitas Indonesia, Jl. Salemba Raya 6, Jakarta, Indonesia; ^7^Faculty of Pharmacy and Sciences, Universitas Muhammadiyah Prof. HAMKA, Jakarta, Indonesia; ^8^Department of Medical Chemistry, Faculty of Medicine, Universitas Indonesia, Jl. Salemba Raya 6, Jakarta, Indonesia

## Abstract

**Background:**

Colorectal cancer (CRC) is a malignancy derived from the glandular epithelial cells in the colon. Patients with inflammatory bowel disease (IBD) are more likely to develop CRC. Cancer proliferation is characterized by the loss of inhibition of apoptosis, which involves caspase-3 activation. This study examined the effects of the pomegranate peel extract on the expression of caspase-3 in mice crypt cells induced by dextran sodium sulfate (DSS) 2%.

**Methods:**

The experimental study was done in six groups. All treatments were done in 42 days. The groups were all induced by DSS through water drinking, except for the normal group, which was only given water. The treatments given included the pomegranate extract in two doses (240 mg and 480 mg/kg bw/day), aspirin, and ellagic acid. The specimens were then fixated and stained for the immunohistochemistry scoring for the expression of caspase-3, which was then analyzed statistically.

**Results:**

The H-scores of each treatment group were 213.23 ± 8.32 (DSS group), 243.81 ± 18.69 (normal group), 226.10 ± 12.38 (pomegranate peel extract of 240 mg/kg/d), 238.84 ± 15.81 (pomegranate peel extract of 480 mg/kg/d), 227.47 ± 12.15 (aspirin), and 224.01 ± 18.39 (ellagic acid). Statistical differences were found in one-way analysis of variance (ANOVA) and *post hoc* analysis among the DSS group, normal group, and dose 2 group (pomegranate peel extract of 480 mg/kg/day).

**Conclusions:**

The ethanol extract of pomegranate was able to induce apoptosis, which was demonstrated by the increase of caspase-3 expression.

## 1. Introduction

Colorectal cancer (CRC), a malignancy also known as colorectal adenocarcinoma, is derived from the glandular epithelial cells in the colon [1]. CRC is a cancer with one of the highest rates of mortality [1, 2]. Inflammatory bowel diseases (IBDs) are known to increase the risk of developing CRC. Although no known genetic factor is able to explain the association, chronic inflammation of the colonic mucosa offers a more likely explanation of the association as patients with a longer duration of colitis have a higher risk of having CRC [3, 4]. The cancer proliferation is characterized by the loss or inhibition of apoptosis [5]. In normal cells, apoptosis is important to balance cell proliferation in the basal layer [6]. Cell apoptosis is divided into two pathways: intrinsic and extrinsic pathways. Both of the apoptosis pathways will equally induce the activation of caspase-3, which further initiates cell apoptosis [7]. The role of caspase-3 in initiating apoptosis makes it a potent target in CRC treatment.

It has been discussed how chronic inflammation of the colon may lead to the development of CRC [7]. Current IBD treatments include anti-inflammatories, some of which are 5-aminosalicylates (5-ASA), corticosteroids, and immunosuppressive or immunomodulatory agents [8]. However, the use of 5-ASA might induce some side effects such as headache, nausea, dyspepsia, diarrhea, pancreatitis, and cholestatic hepatitis [9]. In preventing CRC, aspirin is also associated with a lower risk of incidence and a lower risk of cancer death after five years, including CRC [10]. The use of aspirin, however, has some side effects which include gastrointestinal upsets ranging from nausea and gastritis to gastrointestinal bleeding [11]. In preventing the development of CRC, active compounds such as ellagic acid have been mentioned to have an anti-inflammatory role which can treat ulcerative colitis. Ellagic acid is highly abundant in pomegranates and grapes [12].

Pomegranate (*Punica granatum*) is a shrub of the family Lythraceae [13] commonly found in Iran, northern India, China, and the United States and along the Mediterranean region [14]. The extract of pomegranate is found to have several effects, such as antimicrobial [15], antioxidative, anti-inflammatory, antiangiogenetic, induction of apoptosis, anticancer, and antimutagenic [14]. The anti-inflammatory, antiproliferative, and antitumorigenic effects of the pomegranate extract that work by modulating various cell signaling pathways make it potent as a possible cancer treatment [13]. However, there have been very few studies explaining the effects of the pomegranate peel extract in increasing caspase-3 expression in mice colons. This study examined the effects of the pomegranate peel extract on the expression of caspase-3 in mice after the administration of the pomegranate extract. A significant effect on the expression of caspase-3 by the extract of pomegranate can be further developed for the treatment of CRC.

## 2. Materials and Methods

### 2.1. Study Design and Ethical Clearance

This study was an *in vivo* study that was conducted on 12–16-week-old male Swiss Webster mice weighing about 20–30 g. The mice were bred in the Research and Development Institute of Health (LITBANGKES). Mice were kept under standard laboratory conditions with a temperature of 22°C and humidity of 65%. A diet of standard rodent pellets and water was given *ad libitum* during the treatment. Thirty-three mice were randomly classified into six treatment groups consisting of five to six mice each. The sample size was determined from the Federer formula (*t* – 1)(*n* – 1) ≥ 15 with group numbers (*t*) of 6 and the minimum sample size for each group (*n*) calculated to be ≥4. The experiment was conducted at the Department of Anatomic Pathology, Faculty of Medicine, Universitas Indonesia, Jakarta, Indonesia, from August 2016 to May 2017. Ethical approval of this experiment was granted by the Institutional Animal Ethics Committee of Universitas Indonesia.

### 2.2. Drugs and Chemicals

Ellagic acid was purchased from Santa Cruz Biotechnology (Dallas, TX, USA). Aspirin was purchased from PT Bayer Indonesia. Dextran sodium sulfate (DSS) with a molecular weight of 60 kDa was purchased from Sigma-Aldrich (St. Louis, MO, USA). The caspase-3 primary antibodies were purchased from Abcam. Dosage calculation in this experiment referred to a study conducted by Kusmardi et al. [16]. The dose of pure ellagic acid was determined by a conversion in reference to the United States Food and Drug Administration (FDA) with a dose of 26 mg/kg/day. The dose of aspirin used in this study was 43 mg/kg/day which was calculated from a dose conversion to a mice dose in inducing an anti-inflammatory effect [17].

The doses of the pomegranate peel extract were calculated by determining the compound of ellagic acid in pomegranates using high-performance liquid chromatography (HLPC). The abundance level of ellagic acid in pomegranates was found to be 240 mg/kg/day which then became the reference to the dose administration of the pomegranate peel extract in this study. The second dose of the pomegranate peel extract was 480 mg/kg/day which was obtained by geometric sequence.

### 2.3. Measurement of Ellagic Acid Levels by HPLC

The extract dose was determined based on research by a conversion in reference to the United States Food and Drug Administration (FDA) in rats with an effective anti-inflammatory dose of ellagic acid of 26 mg/kg bw. Levels of ellagic acid compounds were measured by HPLC under the following conditions. (1) Column type: Agilent Zorbax SB-C18 4.6 × 150 mm × 3.5 m; (2) detector: UV 254 nm; (3) flow rate: 1 ml/min; (4) solvent: methanol-water combination. The measurement of ellagic acid levels in the test drug samples begins with the determination of the standard compound curve first. From each concentration of standard ellagic acid solution (25–250 ppm), a curve was obtained which stated the peak position of the ellagic acid compound at a certain retention time. The highest area values for each concentration of the standard solution were then made a standard curve using the linear regression equation *y* = *a*x + *b* in the Microsoft Excel^®^ program. The test extract was carried out with the same procedure as the standard compound solution. The concentration of the pomegranate extract (EP) tested on HPLC was 2000 ppm. The ellagic acid content was calculated by entering the highest area value into the regression equation. The calculation result is the percentage of the ellagic acid content in the test drug sample.

From the concentration of ellagic acid (EA) obtained by HPLC, the levels of ellagic acid in the pomegranate extract (%EA) are as follows:

  %EA = (EAHPLC : EP) × 100
  % EA = levels of ellagic acid in the pomegranate extract
  EAHPLC = the concentration of ellagic acid measured by HPLC (ppm)
  EP = pomegranate extract concentration (ppm)

### 2.4. Experimental Design

The study was done in six treatment groups over a period of 3 × 14 days of treatment (42 days). All treatments were given orally through drinking. In the normal group, mice were given only water orally throughout the experiment. The rest of the groups were given water with 2% DSS for seven days at the start of the second, fourth, and sixth weeks. The administration of DSS was done with an interval of water drinking for 7 days. The negative control group was not given any other treatment other than DSS. Two groups were given pomegranate peel extract in two different doses for each group at the start of the first week of the experiment. Aspirin and pure ellagic acid were given to the other two groups at the start of the first week. All the groups of mice were sacrificed three days after the last treatment of DSS to obtain the colons, which were then cleansed and rinsed with water. Tissues obtained for analysis were colon tissues from proximal to distal with a thickness of 3 mm. The specimens were fixated using 10% buffered neutral formaldehyde (BNF).

### 2.5. Specimen Preparation

After the tissue is fixed, formalin-fixed paraffin-embedded (FFPE) were prepared according to the standard procedure in the pathology laboratory. The FFPE was sliced 3 *μ*m using a microtome and put into the poly-D-lysine-coated slides, warmed, and underwent deparaffinization process according to the standard laboratory procedure.

### 2.6. Immunohistochemistry (IHC) Analysis

The specimens were given immunochemical staining to observe the expression of caspase-3. The observation and IHC analysis in this experiment focused on the nucleus of mice crypt cells. Cells with positive caspase-3 expression would appear with a yellowish-brown nucleus, whereas cells with the negative expression of caspase-3 would appear bluish at the nucleus.

The calculation of caspase-3 expression was done using a histology score (H-score) which was determined using an IHC measurement application, ImageJ 1.53 m. Through microscopic observation, five power fields were randomly taken at a magnification of 400x. Based on the intensity of the color, the score could range from 0, 1, 2, or 3, which represented the negative, weak, medium, and strong color intensity levels. The calculation of the H-score can be summarized in the following formula [16]:

(1) $$\begin{array}{c} \text{H}-\text{score}=\left(\%\text{ cells with negative expression}\times 0\right)\\ +\left(\%\text{ cells with weak positive expression}\times 1\right)\\ +\left(\%\text{ cells with moderate positive expression }\times \;2\right)\\ +\;\left(\%\text{ cells with strong positive expression}\times 3\right). \end{array}$$

### 2.7. Statistical Analysis

Data were analyzed using one-way analysis of variance (ANOVA), followed by Duncan's *post hoc* analysis. The value of *p* < 0.05 indicated a significant difference. The overall statistical analysis of this study was conducted using IBM^®^ SPSS^®^ Statistics 22 software (IBM, Armonk, NY, USA).

## 3. Results

### 3.1. The Results of Measuring the Levels of Ellagic Acid Compounds

**Figure 1:**
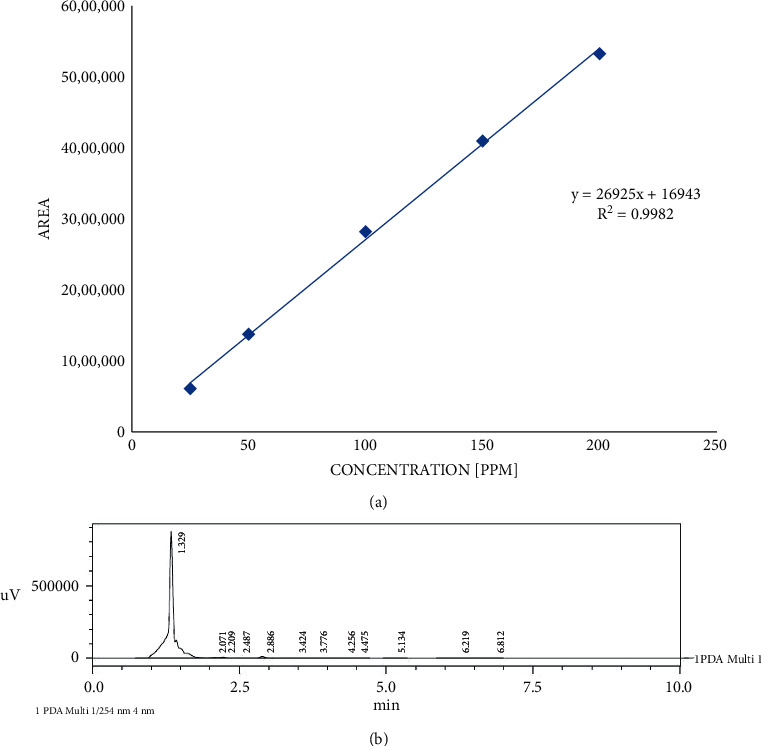
Standard ellagic acid concentration 200 ppm (a) and standard curves of pure ellagic acid of various concentrations (b).

The results of the measurement of ellagic acid in the ethanolic extract of pomegranate peel obtained an area value of 5.898. This value was then entered into the regression equation *y* = 26925*x* + 16943 so that the ellagic acid content of 208.432 ppm or 11.047% is obtained. The regression equation was obtained from the results of processing the chromatogram data for the area of 5 concentrations of pure ellagic acid compounds, namely, 25, 50, 100, 150, and 200 ppm. The results of the area chromatograms for each pure ellagic acid concentration and standard curves are presented in Figure 1.

**Figure 2:**
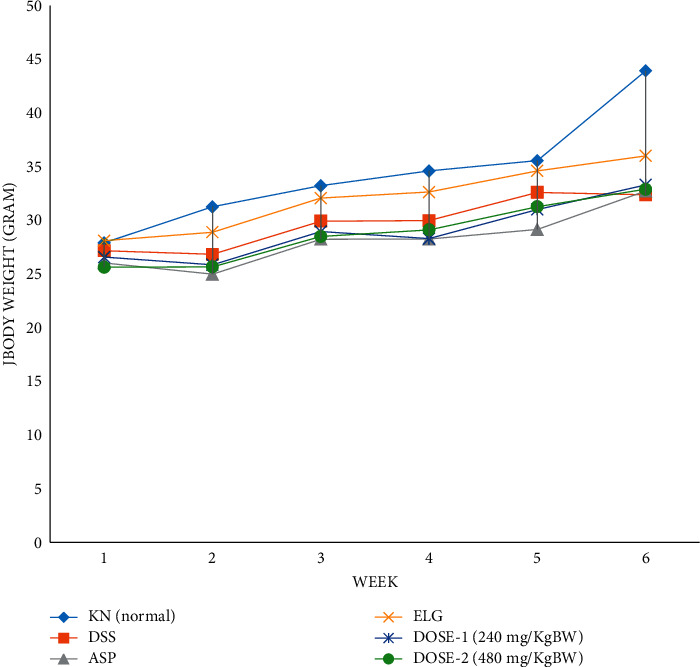
Changes in body weight in various treatment groups were measured per week. KN was normal, DSS group was negative control, Asp was aspirin treated which was a positive control, ELG was the group treated with ellagic acid, dose 1 was the pomegranate peel extract of 240 mg/kg/day, and dose 2 was the pomegranate peel extract of 480 mg/kg/day.

From the determination of the levels of ellagic acid in the extract by HPLC, the levels of ellagic acid in the pomegranate extract (%EA) = (216.7 : 2000) × 100 = 11.047%. It is known from the literature that the effective anti-inflammatory dose of ellagic acid is 26 mg/kg bw. So, when converted to the pomegranate extract, the extract dose (in mg/kg bw) used in this study was = (26 : 11,047) × 100 = 240 mg/kg bw. Changes in body weight in various treatment groups measured per week are shown in Figure 2.

### 3.2. Immunohistochemistry for the Expression of Caspase-3

**Figure 3:**
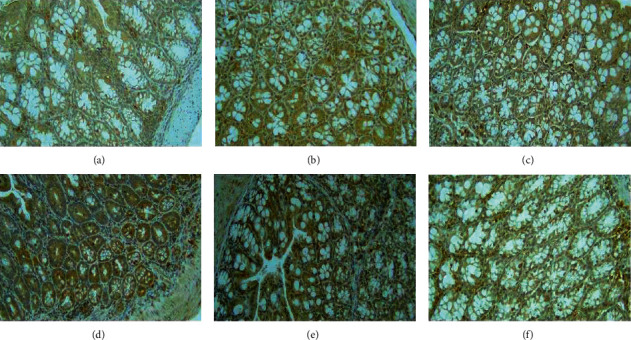
Observation of IHC staining for caspase-3 expression in mice crypt cells. Magnification: ×400. (a) DSS group, (b) normal group, (c) dose 1 (pomegranate peel extract, 240 mg/kg/day), (d) dose 2 (pomegranate peel extract, 480 mg/kg/day), (e) aspirin (43 mg/kg/day), and (f) ellagic acid (26 mg/kg/day).

In the microscopic observation of immunostaining, Figure 3 shows that ethanol extracts of pomegranate peel (dose 1 and dose 2), aspirin, and ellagic acid increased the expression of caspase-3, which is shown by the positive intensity of the staining in the brown-stained cells. In comparison with the negative control group, the weak intensity of the brown-stained and some blue-colored cells can also be seen, which indicates low to no expression of caspase-3. Quantitative measurements of caspase-3 expression were done using the H-score.

**Figure 4:**
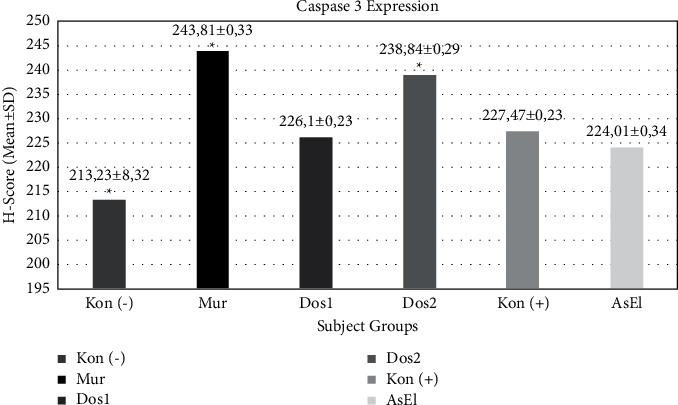
The caspase-3 expression in mice crypt cells is indicated by the H-score. Data are presented in the form of mean ± standard deviation (*N* = 33). Kon(−) was the DSS group, Mur was the normal group, Dos 1 was dose 1: pomegranate peel extract of 240 mg/kg/day, dose 2 (Dos 2) was the pomegranate peel extract of 480 mg/kg/day, Kon (+) was aspirin, and AsEl group was treated with aspirin and pure ellagic acid. ^*∗*^Significant differences with the DSS group (*p* < 0.05); ^*∗∗*^no significant differences among dose 1, ASP, and ELA. DSS group, (NORM): normal group, dose 1 (Dos 1): pomegranate peel extract of 240 mg/kg/day, dose 2 (DOS2): pomegranate peel extract of 480 mg/kg/day, ASP: aspirin, and ELA: pure ellagic acid.

The results of the one-way ANOVA are shown on the chart in Figure 4. The mean H-scores obtained for each treatment group were 213.23 ± 8.32 (DSS group), 243.81 ± 18.69 (normal group), 226.10 ± 12.38 (pomegranate peel extract of 240 mg/kg/day), 238.84 ± 15.81 (pomegranate peel extract of 480 mg/kg/day), 227.47 ± 12.15 (aspirin), and 224.01 ± 18.39 (ellagic acid). The data collected had a normal distribution with a homogeneous variance. The one-way ANOVA test showed significant differences (*p*=0.023), and Duncan's *post hoc* analysis showed significant differences between the negative control group and the normal group and the negative control with dose 2 (pomegranate peel extract of 480 mg/kg/day).

## 4. Discussion

In this study, we found that the ellagic acid concentration in our extract was approximately 11% and was estimated as an active compound in the pomegranate peel extract. Therefore, the doses that were given to the mice adjusted the concentrations of the extract as the recommendation calculation of the FDA. Interestingly, in this study, we show in Figure 2 that no weight loss in the group that received the extract was observed, while weight loss at the other groups was observed.

Moreover, the results of this study show that the ethanol extract of pomegranate peel can increase the expression of caspase-3 in DSS-induced mice. The expression of caspase-3 in mice crypt cells increased as the dose of the extract increased. The increased level of caspase-3 expression by the extract in this study exceeded the caspase-3 expression of aspirin and pure ellagic acid. Caspase-3 expression of the dose 2 extract was even closer to the caspase-3 expression of the normal group, which represents a normal level of apoptosis. DSS is a sulfated polysaccharide that is water soluble and negatively charged. DSS is commonly used in mice to induce inflammation in the colon by causing damage to the epithelial layer, thereby releasing the proinflammatory cytokines from the tissues underneath [18]. Mice models with the induction of DSS are commonly used for conducting studies related to inflammatory bowel disease (IBD) [18, 19]. Aspirin was used as a positive control in this study. Aspirin, as one of the types of nonsteroidal anti-inflammatory drugs (NSAIDs), has been found to induce apoptosis in CRC cells [20]. Increased expression of caspase-3 was found in another study conducted by Yan et al. [21] occurring through AP-2*α* degradation. Ellagic acid is an active substance that was used as an active control in this study. Ellagic acid, one of the phenol substances, is known to emit a cellular response, one of which is apoptosis through a cascade of molecular activations. The study conducted by Li et al. [22] found that ellagic acid promoted the expression of caspase-3, thereby inducing apoptosis in T24 bladder cancer cells.

The significant increase of caspase-3 expression in this study is similar to the study conducted by Shaban et al., showing similar results in which there was an increase in the expression of caspase-3 in the mice given the pomegranate peel extract compared with a group of mice that were not given the treatment [23]. The study conducted by Deng et al. determined that the pomegranate extract increased the expression ratio of Bax/Bcl-2 and promoted the activation of caspase-3 as an apoptosis executor [24]. Similar effects on the increased expression of caspase-3 were also found in studies conducted by Sepehr Sineh et al. [25] and Bishayee et al. [26]. In addition to the increased expression of caspase-3, the extract of pomegranate peel also enhanced the expression of caspase-9 in a study conducted by Abdul Ghani et al. The study concluded that the pomegranate peel extract induced apoptosis through the mitochondrial pathway [27].

Pomegranate is known to have anticancer effects that are obtained from its high content of polyphenols. The anticancer effects of pomegranate result from its antiproliferative, anti-invasive, and antimetastatic qualities. Apoptosis infusion effects are also activated by pomegranate through its modulation of Bcl-2, upregulation on p21 and p27, and the downregulation of the cyclin-CDK tissue [28]. Amer et al. demonstrated pomegranate is also known to have activity to suppress reactive oxygen species which is needed in the activation of inflammatory processes, especially suppressing cell proliferation and activation of immune cells. Thus, the anti-ROS activity of pomegranate however is used to reduce inflammation and also reduce cell death due to oxidative stress [29].

Although the method in this experiment was to use pomegranate peel extract as a prevention of CRC development after IBD, these results showed the potency of the extract to be developed as curative therapy as well. The ability of pomegranate in enhancing the expression of caspase-3 as an apoptosis executor makes it a potent substance to be further studied as an anticancer drug with caspase-3 as the molecular target.

## 5. Conclusions

Caspase-3, which plays an important role in apoptosis induction, shows potency as a target in anticancer therapy. Extract of pomegranate peel with high levels of polyphenols is known to have anticancer effects, one of which is through apoptosis induction, which, in this study, was demonstrated with the increased expression of caspase-3. Further studies are still required to explore the efficacy and safety of the pomegranate peel extract in inducing apoptosis as a way to prevent CRC development from IBD.

## Data Availability

The data used to support the findings of this study are available from the corresponding author upon request.
